# Venereal Diseases among Seamen

**Published:** 1920-08-14

**Authors:** 


### Venereal Diseases among Seamen.

The International Labour Conference established under the Treaty of Peace has been recently sitting for three weeks at Genoa for the purpose of improving by national legislation, based on international agreements, the lot of the seafaring man. The main question before the Conference, and the most contentious, was that of the eight-hour day for seamen. The delegates, however, found time to give attention to some aspects of the health of seamen, and notably the question of the prevention and treatment of venereal diseases among those who "go down to the sea in ships."

At the first sitting of the Conference an invitation to the delegates (who represent the Governments, shipowners and seamen of a score of countries) to attend a meeting on the question of combating venereal diseases in the mercantile marine was communicated to the Conference in the names of Mrs. A. C. Gotto and Dr. Otto May, representatives of the National Council for Combating Venereal Disease. As a result of this meeting, Mr. C. Hipwood (Assistant Secretary, Board of Trade), one of the British Government delegates, wrote to M. Albert Thomas, Director of the International Labour Office, stating that a strong hope had been expressed at the meeting that the Conference would not disperse without taking some definite action to help on the work of fighting these diseases. Mr. Hipwood added that a small committee of representatives of the National Council, seamen, shipowners and Governments, had prepared the following resolution, which they hoped the Conference would be willing to consider:

"The International Seamen's Conference, recognising the importance of taking active international measures for the prevention and treatment of venereal diseases in the mercantile marine, desires to urge upon the Health Section of the League of Nations the need for immediate attention to this subject.

"They would recommend for special consideration: (1) The provision of adequate facilities for the prevention and treatment of venereal diseases at all the principal ports. (2) The inclusion of venereal diseases among the conditions for which 'Free' drugs and treatment are provided for members of the mercantile marine. (3) The dissemination of appropriate information on the subject to seafarers, especially to those at training establishments; and (4) The provision of adequate facilities for recreation at all large ports under the administration of joint organisations representative of owners and seafarers.

"They desire, in addition, to call the special attention of the International Labour organisations to the importance of No. 4 about facilities for recreation."

According to an unofficial report received from Genoa a few days ago, this resolution was adopted by the Conference.

